# Spinal PAR2 Activation Contributes to Hypersensitivity Induced by Peripheral Inflammation in Rats

**DOI:** 10.3390/ijms22030991

**Published:** 2021-01-20

**Authors:** Petra Mrozkova, Diana Spicarova, Jiri Palecek

**Affiliations:** Laboratory of Pain Research, Institute of Physiology, Czech Academy of Sciences, Videnska 1083, 142 20 Prague 4, Czech Republic; Petra.Mrozkova@fgu.cas.cz (P.M.); Jiri.Palecek@fgu.cas.cz (J.P.)

**Keywords:** PAR2, TRPV1, synaptic transmission, superficial dorsal horn, spinal cord, nociception, peripheral inflammation, thermal hyperalgesia, inflammatory pain

## Abstract

The mechanisms of inflammatory pain need to be identified in order to find new superior treatments. Protease-activated receptors 2 (PAR2) and transient receptor potential vanilloid 1 (TRPV1) are highly co-expressed in dorsal root ganglion neurons and implicated in pain development. Here, we examined the role of spinal PAR2 in hyperalgesia and the modulation of synaptic transmission in carrageenan-induced peripheral inflammation, using intrathecal (i.t.) treatment in the behavioral experiments and recordings of spontaneous, miniature and dorsal root stimulation-evoked excitatory postsynaptic currents (sEPSCs, mEPSCs and eEPSCs) in spinal cord slices. Intrathecal PAR2-activating peptide (AP) administration aggravated the carrageenan-induced thermal hyperalgesia, and this was prevented by a TRPV1 antagonist (SB 366791) and staurosporine i.t. pretreatment. Additionally, the frequency of the mEPSC and sEPSC and the amplitude of the eEPSC recorded from the superficial dorsal horn neurons were enhanced after acute PAR2 AP application, while prevented with SB 366791 or staurosporine pretreatment. PAR2 antagonist application reduced the thermal hyperalgesia and decreased the frequency of mEPSC and sEPSC and the amplitude of eEPSC. Our findings highlight the contribution of spinal PAR2 activation to carrageenan-induced hyperalgesia and the importance of dorsal horn PAR2 and TRPV1 receptor interactions in the modulation of nociceptive synaptic transmission.

## 1. Introduction

Cloning of the seven-transmembrane G protein-coupled receptor (GPCR) protease-activated receptor 2 (PAR2) [1] allowed a detailed investigation of its role in a number of physiological and pathophysiological processes, including acute and chronic inflammation and hypersensitivity induction [2,3,4,5,6,7]. Numerous endogenous proteases target PAR2 to cleave the extracellular amino terminus at canonical cleavage sites (trypsin and mast cell tryptase) [8] to activate canonical pathways of GPCR signaling or at distinct sites (neutrophil elastase or macrophage cathepsin S) to activate the biased mechanisms [7,9,10]. Several synthetic PAR2-activating peptides (PAR2 AP) were developed, and in our experiments, we used SLIGKV-NH2, which corresponds to the tethered ligand domain and mimics the canonical mechanism of activation by the endogenous activators [11,12]. Proteolytic cleavage at the canonical sites of PAR2 induces both signaling at the cell membrane and, also, endosomal signaling pathways [7,13,14]. Signaling from the plasma membrane involves the phospholipase C (PLC)-dependent production of inositol trisphosphate (IP_3_) and diacylglycerol (DAG), followed by an IP_3_-stimulated release of Ca^2+^ from the intracellular storage, which, together with DAG, leads to protein kinase C (PKC) activation [15,16,17]. Signaling from the endosome requires PAR2 association with β-arrestin, followed by endocytosis. Sustained endosomal signaling initiates mitogen-activated protein kinase ERK1/2 activation and gene transcription [7,14,18,19]. The biased mechanism of PAR2 activation induces signaling from the plasma membrane to activate adenylyl cyclase, resulting in the activation of PKA, while it does not involve a β-arrestin interaction or endocytosis of the receptor [7,20].

PAR2s are widely distributed in both the peripheral and central nervous systems. About 60% of neurons in the L4–L6 dorsal root ganglia (DRG) express PAR2 [21]. Most of the DRG neurons in the culture responded to PAR2 AP, and all of the neurons responding to trypsin were capsaicin-sensitive [15], indicating an extremely high co-expression of PAR2 with transient receptor potential vanilloid 1 (TRPV1) receptors, one of the key elements involved in nociception [22,23]. This functional evidence corresponds well with the expression of PAR2 in ~ 90% of TRPV1-positive DRG neurons, whereas ~60% of PAR2-expressing DRG neurons were TRPV1-positive [16]. In agreement with the high co-expression of both receptors in the DRG neurons, the modulation of TRPV1 by PAR2 activation was demonstrated in vitro. The activation of PAR2 increased the capsaicin-evoked TRPV1 currents, increased the intracellular Ca^2+^ concentration and decreased the temperature thresholds for TRPV1 activation from 42 °C to below the body temperature [16,24]. The sensitization of TRPV1 induced by PAR2 activation is mediated by PLCβ, PKCε and PKA signaling [24], similar to the increased sensitivity of spinal TRPV1 to endogenous agonists by proinflammatory cytokines [25,26,27]. The role of PAR2 in nociception is further substantiated by the release of substance P and calcitonin gene-related peptides from both the peripheral and central endings of the DRG neurons after PAR2 activation [5]. The activation of spinal PAR2 also induced prostaglandin E2 release in the spinal cord tissue [28].

The activation of PAR2 in DRG neurons stimulates PLC and induces PK activation that modulates the activity of TRPV1, TRPV4, TRPA1 [16,24,29,30,31], voltage-gated Kv7 potassium and calcium-activated Cl^−^ channels [17,32,33]. This regulates the excitability of these primary nociceptive neurons, neuropeptide releases [5] and synaptic transmissions at the first nociceptive synapse in the spinal cord dorsal horn and leads to hypersensitivity in vivo [34,35]. The involvement of TRPV1 in hypersensitivity induced by an intraplantar injection of PAR2 AP was demonstrated by a subcutaneous pretreatment with a TRPV1 antagonist [15] and genetic deletion of the TRPV1 channel [15,16]. Hypersensitivity can be also induced by central mechanisms after an intrathecal (i.t.) treatment with the PAR2 agonist [21,34,36]. This spinal PAR2 activation-induced hypersensitivity was mediated by TRPV1 and did not develop in PAR2 knockout animals [21,34]. Thus, the hypersensitivity elicited by PAR2 activation via TRPV1 could be mediated by both the peripheral and spinal mechanisms.

The pronociceptive role of PAR2 was investigated in a variety of inflammatory models, where increased cytokines levels and PAR2 expression were demonstrated in accordance with the in vitro studies [2]. However, in PAR2-deficient mice, the inflammatory cytokines and other signs of inflammation are suppressed [37,38,39]. In the model of rheumatoid arthritis induced by a mixture of carrageenan and kaolin or complete Freund’s adjuvant (CFA), the PAR2 expression was increased in the synovium and the periarticular tissues, while the joint inflammation was attenuated in the PAR2-deficient mice [3,40]. Hypersensitivity was also attenuated in PAR2 knockout mice after an intraplantar injection of formalin [41].

In the present study, we investigated the role of spinal PAR2 in behavioral hypersensitivity and in modulation of the synaptic transmission in the superficial dorsal horn after the induction of peripheral inflammation by intraplantar carrageenan injection. The involvement of TRPV1 receptors and protein kinase activation were also evaluated.

## 2. Results

### 2.1. Activation of Spinal PAR2 in Thermal Hypersensitivity Induced by Peripheral Inflammation

The role of spinal PAR2 in thermal hypersensitivity after the induction of peripheral inflammation was examined in in vivo experiments. The paw withdrawal latency (PWL) in response to a thermal stimulus decreased 24 h after the intraplantar carrageenan injection to 57.83% ± 3.5%, *n* = 4, *p* < 0.001). This decrease was robust and stable even 32 h after the injection (56.8% ± 4.2%, *n* = 4, *p* < 0.001). Thus, the decrease of PWL 24 h after the carrageenan injection was considered as fully developed inflammation and taken as a control value (ctrl 100%) just prior to the intrathecal administration of the tested compounds.

**Figure 1:**
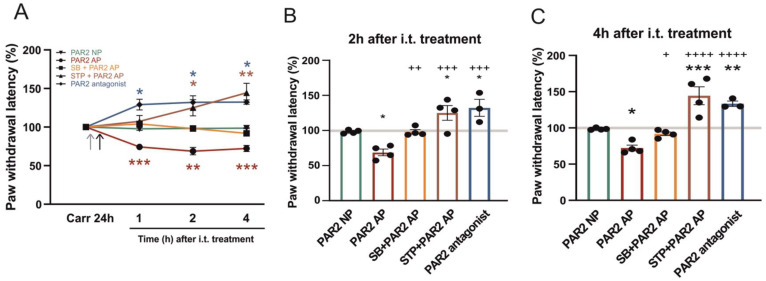
The effect of spinal protease-activated receptor 2 (PAR2) activation and inhibition on carrageenan-induced thermal hyperalgesia. (**A**) Thermal hypersensitivity measured 24 h after the carrageenan injection, just before the intrathecal (i.t.) treatments were considered as 100%. The administration of the inactive reverse peptide PAR2 NP (*n* = 4) did not change the thermal threshold any further. In comparison, PAR2-activating peptide (AP) administration (*n* = 4) decreased the paw withdrawal latency (PWL) to the radiant heat stimulation for several hours. The transient receptor potential vanilloid 1 (TRPV1) antagonist SB 366791 (SB, *n* = 4) pretreatment prevented the PAR2 AP-induced decrease of PWL. Staurosporine (STP, *n* = 4) pretreatment also prevented the PAR2 AP-induced effect, and the thermal threshold even increased (2 h and 4 h). PAR2 antagonist (*n* = 3) administration increased the PWL for several hours (1 h, 2 h and 4 h). Grey arrowhead: SB 366791 or staurosporine i.t. pretreatment. Black arrowhead: PAR2 NP, PAR2 AP or PAR2 antagonist i.t. treatment. Statistical differences were identified using Kruskal-Wallis one-way ANOVA for each group; * *p* < 0.05, ** *p* < 0.01 and *** *p* < 0.001, comparison with the control before i.t. treatment. (**B**,**C**) A detailed comparison of various treatments at 2 and 4 h after the i.t. treatment. Statistical differences between groups with various treatments were identified using one-way ANOVA followed by the Student-Newman-Keuls (SNK) test; * *p* < 0.05, ** *p* < 0.01 and *** *p* < 0.001, comparison with PAR2 NP and + *p* < 0.05, ++ *p* < 0.01, +++ *p* < 0.001 and ++++ *p* < 0.0001, comparison with PAR2 AP.

In the control experiments, a PAR2 nonactive reverse peptide (PAR2 NP, VKGILS-NH2) was tested in a group of four animals. The PWLs decreased to 57.1% ± 3.5% (*n* = 4, *p* < 0.001) 24 h after the carrageenan injection. This decrease was further considered as 100% just before the i.t. treatment. The administration of PAR2 NP (8 μg in 10 μL of saline, i.t.) did not further change the PWL at any of the tested time points (one hour after the i.t. treatment, 97.82% ± 5.1%; two hours, 98.30% ± 4.8% and four hours, 98.57% ± 4.1%, Figure 1A). The thermal hyperalgesia induced by intraplantar carrageenan treatment was not affected by the intrathecal administration of PAR2 NP.

In the experimental group of animals, the effect of the PAR2-activating peptide (PAR2 AP, SLIGKV-NH_2_) was evaluated. A carrageenan injection into the rat paw decreased the PWLs to 63.5% ± 3.0% (*n* = 4, *p* < 0.001), which were considered 100% before the i.t. treatment. The subsequent administration of PAR2 AP (8 μg in 10 μL of saline, i.t.) decreased the PWL already one hour after the treatment (74.3% ± 1.8%, *n* = 4, *p* < 0.001, Figure 1A). This decrease of PWL lasted and was even more pronounced at two hours and four hours after the PAR2 AP administration (two hours, 68.8% ± 4.8%, *p* < 0.01 and four hours, 72.2% ± 4.0%, *p* < 0.001). A PAR2 AP-induced decrease of the PWL was also statistically significant when compared to the PAR2 NP i.t. treatment (Figure 1B,C). The activation of spinal PAR2 further increased the thermal hyperalgesia above the carrageenan-induced inflammation alone.

Another set of behavioral experiments was performed to test the role of spinal TRPV1 receptors in PAR2 activation-induced hyperalgesia under inflammatory conditions. The carrageenan injection decreased the PWLs to 60.5% ± 3.0% (*n* = 4, *p* < 0.001), which was considered as 100% for further evaluation after the i.t. treatment. The administration of SB 366791 (0.43 μg in 15 μL of saline, i.t.) 10 min before the PAR2 AP dose (8 μg in 10 μL of saline, i.t.) prevented any further change of the PWL (one hour, 104.5% ± 3.9%; two hours 98.1% ± 3.0% and four hours, 91.9% ± 2.5%; *n* = 4, Figure 1). In the experiments studying the involvement of PK activation in the PAR2-induced hyperalgesia carrageenan injection decreased the PWLs to 57.3% ± 1.7% (*n* = 4, *p* < 0.001), considered as 100% for the subsequent i.t. treatment. A broad-spectrum PK inhibitor, staurosporine (0.014 μg in 15 μL of saline, i.t.), was administered 10 min before the PAR2 AP (8 μg in 10 μL of saline, i.t.). The PWLs did not change one hour after the i.t. treatment, while they significantly increased two hours and four hours after the treatment (one hour, 107.4% ± 7.6%; two hours, 125.1% ± 10.5%, *p* < 0.05 and four hours, 144.4% ± 12.3%, *p* < 0.01; *n* = 4, Figure 1A). These results indicated that the inhibition of spinal PKs prevented spinal PAR2 activation-induced thermal hyperalgesia and reduced the hypersensitivity caused by the peripheral inflammation.

In the last set of behavioral experiments, we examined the effect of spinal PAR2 activation under inflammatory conditions using the PAR2 peptide antagonist FSLLRY-NH2. The carrageenan injection decreased the PWLs to 50.2% ± 1.3% (*n* = 3, *p* < 0.001), considered as 100% for the subsequent i.t. treatment. Administration of the PAR2 peptide antagonist (10 μg in 10 μL of saline, i.t.) significantly increased the PWL already one hour after the treatment (one hour, 126.0% ± 8.7%, *p* < 0.05). This increase of the PWL lasted and was even more pronounced at two hours and four hours after the administration of the PAR2 antagonist (two hours, 132.3% ± 12.0%, *p* < 0.05 and four hours, 133.3% ± 3.7%, *p* < 0.05, *n* = 3, Figure 1A). These results indicated that the activation of spinal PAR2 mediated at least part of the thermal hyperalgesia associated with the peripheral inflammation.

### 2.2. Modulation of Miniature Excitatory Postsynaptic Currents (mEPSCs) in Dorsal Horn Neurons by PAR2 under the Inflammatory Conditions

The modulation of the mEPSCs recorded from the superficial dorsal horn neurons after PAR2 activation and inhibition was tested in vitro using spinal cord slices dissected 24 h after the carrageenan injection in the hind paw. Miniature EPSCs were recorded in 37 neurons, where the average control mEPSC frequency was 1.13 ± 0.10 Hz. In 35 neurons out of these, capsaicin (0.2 μM) applied at the end of the recording induced an increase of the mEPSC frequency. This suggests the presence of presynaptic TRPV1 receptors localized on the central endings of these DRG neurons.

**Figure 2:**
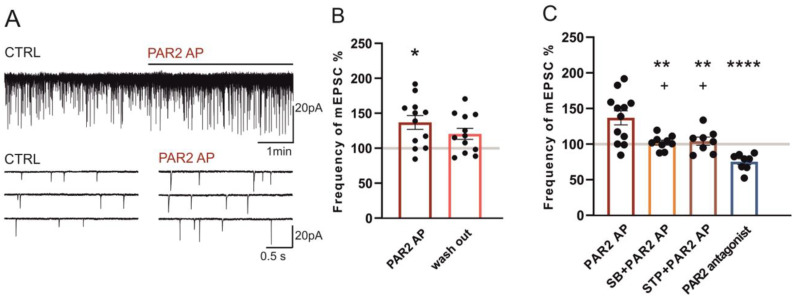
Activation of PAR2 increased the frequency of the miniature excitatory postsynaptic currents (mEPSCs) under inflammatory conditions. (**A**) Application of PAR2 AP (4 min) on a spinal cord slice prepared 24 h after the carrageenan injection increased the frequency of the mEPSC recorded in the superficial dorsal horn neuron. (**B**) Application of PAR2 AP increased the average mEPSC frequency (*n* = 12; Kruskal-Wallis one-way ANOVA, * *p* < 0.05), with a partial decline during the washout period (4 min). (**C**) Pretreatment and co-application of the TRPV1 antagonist SB 366791 (SB, *n* = 9) or staurosporine (STP, *n* = 8) with PAR2 AP prevented the excitatory effect of the PAR2 AP treatment. The application of the PAR2 antagonist (*n* = 8) decreased the average mEPSC frequency. Statistical analysis: one-way ANOVA followed by the SNK test. Statistical symbols: ** *p* < 0.01 and **** *p* < 0.0001 versus PAR2 AP and + *p* < 0.05 versus the PAR2 antagonist.

The application of PAR2 AP (100 µM, 4 min) significantly increased the mEPSC frequency to 136.8% ± 10.0% (*n* = 12, *p* < 0.05, Figure 2A,B). This excitatory effect on the mEPSC frequency declined during the four-minute washout period (120.5% ± 5.5%). The average amplitude of the mEPSCs did not change after the PAR2 AP application (control: 23.1 ± 1.2 pA and PAR2 AP: 21.1 ± 0.8 pA).

The interaction of the PAR2 and TRPV1 receptors under the inflammatory conditions was evaluated in further experiments. The frequency of the mEPSC remained unchanged during the application of the TRPV1 antagonist SB 366791 alone (10 μM, 4 min, 103.2% ± 7.5%, *n* = 9). The subsequent co-application of SB 366791 (10 μM) with PAR2 AP (100 µM, four minutes) also did not change the mEPSC frequency (102.1% ± 3.4%, Figure 2C) when compared to the period of pretreatment with SB 366791. The mean amplitude of the mEPSC did not significantly change throughout the treatments (control: 21.7 ± 2.2 pA, SB 366791: 22.8 ± 2.7 pA and SB 366791/PAR2 AP: 20.4 ± 2.3 pA). These results indicate that, under the inflammatory conditions, PAR2 activation increased the mEPSC frequency in the superficial dorsal horn neurons, and this was mediated by the TRPV1 receptor activation.

The involvement of protein kinases was evaluated in another group of superficial dorsal horn neurons by staurosporine (250 nM, four minutes), which had no effect on the mEPSC frequency when applied alone (97.6% ± 3.2%, *n* = 8). The subsequent co-application of staurosporine (250 nM) with PAR2 AP (100 µM, four minutes) also did not change the mEPSC frequency (104.1% ± 5.7%, *n* = 8, Figure 2C) when compared to the pretreatment with staurosporine alone. The mean amplitude of the mEPSC did not change throughout the treatments (control: 23.9 ± 3.3 pA, staurosporine: 22.6 ± 2.6 pA and staurosporine/PAR2 AP: 24.0 ± 3.8 pA). Staurosporine thus prevented the mEPSC frequency increase mediated by PAR2 activation.

In another group of superficial dorsal horn neurons, the application of the PAR2 peptide antagonist FSLLRY-NH_2_ (100 μM, four minutes) decreased the frequency of the mEPSC (75.2% ± 4.2%, *n* = 8, *p* < 0.01, Wilcoxon signed rank test, Figure 2C). The mean amplitude of the mEPSC did not change throughout the treatments (control: 21.7 ± 1.5 pA and PAR2 antagonist: 22.7 ± 2.1 pA). This suggests that spinal PAR2 receptors are already activated under the conditions of peripheral inflammation.

### 2.3. Modulation of Spontaneous Excitatory Postsynaptic Currents (sEPSCs) in Dorsal Horn Neurons by PAR2 under Inflammatory Conditions

The basal control sEPSC frequency recorded in the dorsal horn neurons under the inflammatory conditions was higher 1.58 ± 0.15 Hz (*n* = 41, *p* < 0.5) than the basal mEPSC frequency (see Section 2.2). Out of these 41 neurons, 39 neurons showed increased frequencies of the sEPSC after the capsaicin (0.2 μM) application at the end of the experimental protocol.

**Figure 3:**
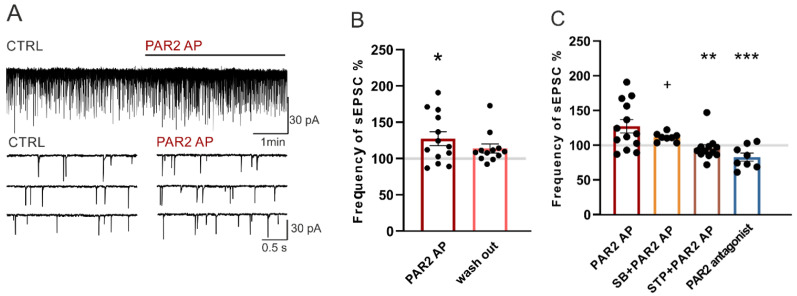
PAR2 activation increased the frequency of the spontaneous excitatory postsynaptic currents (sEPSCs) under the inflammatory conditions. (**A**) Application of PAR2 AP (4 min) on an acute spinal cord slice prepared 24 h after the carrageenan injection increased the sEPSC frequency as documented in the recording from one superficial dorsal horn neuron. (**B**) Application of PAR2 AP increased the average sEPSC frequency (*n* = 13; Kruskal-Wallis one-way ANOVA, * *p* < 0.05). (**C**) Pretreatment and co-application of the TRPV1 antagonist SB 366791 (SB, *n* = 8) attenuated the PAR2 AP-induced effect on the sEPSC frequency. Pretreatment and co-application of staurosporine (STP, *n* = 12) prevented the excitatory effect of PAR2 AP. Application of the PAR2 antagonist (*n* = 8) decreased the frequency of the sEPSC. Statistical analysis: one-way ANOVA followed by SNK test. Statistical symbols: ** *p* < 0.01 and *** *p* < 0.001 versus PAR2 AP and + *p* < 0.05 versus the PAR2 antagonist.

A bath application of PAR2 AP (100 µM, four minutes) significantly increased the sEPSC frequency to 127.2% ± 9.5% (*n* = 13, *p* < 0.05, Figure 3A,B). The excitatory effect of the PAR2 AP application on the sEPSC frequency declined during the four-minute washout period (115.0% ± 9.1%). The average amplitude of the recorded sEPSCs did not change significantly in any of the experimental conditions (control: 22.4 ± 2.1 pA and PAR2 AP: 21.3 ± 1.9 pA).

The application of SB 366791 (10 µM, four minutes) alone did not change the sEPSCs frequencies (102.0% ± 6.1%, *n* = 8) in another set of experiments. The subsequent co-application of SB 366791 (10 µM) with PAR2 AP (100 µM, four minutes) slightly increased the sEPSC frequency (111.2% ± 2.2%, Figure 3C) when compared to the SB 366791 pretreatment period, but this increase was not statistically significant. The average amplitude of the recorded sEPSCs did not change in any of the experimental conditions (control: 22.6 ± 1.9 pA, SB 366791: 22.2 ± 1.3 pA and SB 366791/PAR2 AP: 22.0 ± 1.2 pA).

The staurosporine (250 nM, four minutes) application had no effect on the sEPSC frequency (99.1% ± 4.1%, *n* = 12) in another set of experiments. The subsequent co-application of staurosporine (250 nM) with PAR2 AP (100 µM, four minutes) completely prevented the sEPSC frequency increase present after the PAR2 AP application alone (95.5% ± 5.2%, Figure 3C). The average amplitude of the recorded sEPSCs did not change significantly in any of the experimental conditions (control: 22.8 ± 2.0 pA, staurosporine: 22.4 ± 2.3 pA and staurosporine/PAR2 AP: 22.5 ± 2.2 pA).

PAR2 activation under the inflammatory conditions was tested using an application of the PAR2 peptide antagonist FSLLRY-NH2 (100 μM, four minutes). Application of the PAR2 antagonist decreased the sEPSC frequency to 82.8% ± 3.8% (*n* = 8, *p* < 0.05, Wilcoxon signed rank test, Figure 3C). The average amplitude of the recorded sEPSCs did not change during the experiment (control: 24.1 ± 2.6 pA and PAR2 antagonist: 25.1 ± 2.6 pA). This inhibitory effect of the PAR2 antagonist on the sEPSC frequency is in agreement with the inhibitory effect on the mEPSC frequency and suggests the presence of activated PAR2 receptors.

### 2.4. Modulation of Dorsal Root Stimulation-Evoked EPSC by PAR2 under the Inflammatory Conditions

To record the eEPSCs, the dorsal root attached to the spinal cord slice was electrically stimulated with a glass suction electrode in 30-s intervals. Evoked EPSCs were recorded in 38 neurons; capsaicin (0.2 μM) applied at the end of the experiment induced an increase of the sEPSC frequency in 35 of them.

**Figure 4:**
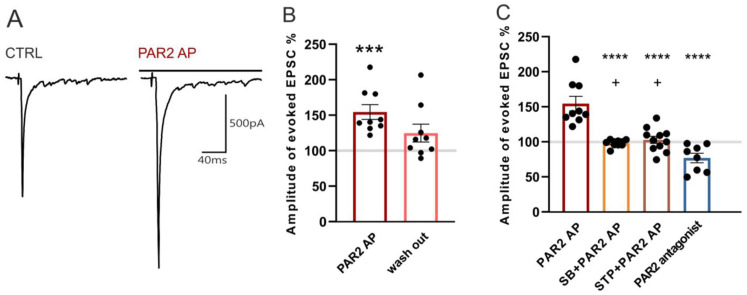
Activation of PAR2 increased the amplitude of EPSCs evoked by dorsal root stimulation. (**A**) Native recordings from one superficial dorsal horn neuron in an acute spinal cord slice prepared 24 h after the carrageenan injection. Application of PAR2 AP (4 min) increased the amplitude of the evoked EPSC. (**B**) The increase of the evoked EPSC (eEPSC) amplitude during the PAR2 AP application was statistically significant (*n* = 9; Kruskal-Wallis one-way ANOVA, *** *p* < 0.001). (**C**) Pretreatment and co-application of SB 366791 (SB, *n* = 10) or staurosporine (STP, *n* = 11) with PAR2 AP prevented the PAR2 AP-induced increase of the eEPSC amplitude. Application of the PAR2 antagonist (*n* = 8) decreased the eEPSC amplitude. Statistical analysis: one-way ANOVA followed by the SNK test. Statistical symbols: **** *p* < 0.0001 versus PAR2 AP and + *p* < 0.05 versus the PAR2 antagonist.

In the first series of experiments, a bath application of PAR2 AP (100 µM, four minutes) increased the amplitude of the evoked EPSCs (154.4% ± 10.4%, *n* = 9, *p* < 0.001, Figure 4A,B), and this increase declined during the subsequent four minutes of the washout period (124.7% ± 12.6%). This increase of the synaptic current amplitude suggests that PAR2 activation leads to enhanced nociceptive synaptic transmission in the superficial spinal cord dorsal horn under inflammatory conditions.

The possible involvement of TRPV1 in the PAR2 activation-induced eEPSC amplitude increase was examined by the application of SB 366791 (10 µM, four minutes) that, alone, had no effect on the mean amplitude (101.7% ± 4.1%, *n* = 10). The subsequent co-application of SB 366791 (10 µM) with PAR2 AP (100 μM, four minutes) also did not change the eEPSC amplitude (98.1% ± 1.6%, Figure 4C) when compared to the SB 366791 pretreatment. The inhibition of the spinal TRPV1 receptors thus prevented the PAR2 activation-induced increase of the eEPSC amplitude.

In another group of neurons, a staurosporine (250 nM, four minutes) application did not change the eEPSC amplitude (93.6% ± 6.3%, *n* = 11). The subsequent co-application of staurosporine (250 nM) with PAR2 AP (100 μM, four minutes) similarly had no effect on the eEPSC amplitude (102.7% ± 5.1%, Figure 4C). The inhibition of PKs thus prevented the increase of the eEPSC amplitude induced by spinal PAR2 activation.

In the last group of superficial dorsal horn neurons, the application of the PAR2 peptide antagonist FSLLRY-NH2 (100 μM, four minutes) decreased the eEPSC amplitude (76.9% ± 6.8%, *n* = 8, *p* < 0.01, Wilcoxon signed rank test), suggesting that the spinal PAR2 receptors were already active after the induction of the peripheral inflammation.

## 3. Discussion

Starting from the demonstration that PAR2 agonists have a role in neurogenic inflammation [5], the significance of PAR2 activation in the nervous system was studied in a variety of pathological pain conditions [42]. In the present study, we investigated the modulation of thermal hypersensitivity and nociceptive synaptic transmission in the spinal cord by the activation and inhibition of spinal PAR2 24 h after the induction of peripheral inflammation. Our in vivo experiments showed that the activation of spinal PAR2 using an i.t. administration of PAR2 AP further aggravated the thermal hyperalgesia associated with the carrageenan-induced peripheral inflammation in adult rats. This spinal PAR2 activation-mediated increase of thermal hypersensitivity was prevented by the inhibition of spinal TRPV1 receptors or protein kinases, whereas the latter also improved the carrageenan-induced hypersensitivity itself. Importantly, the carrageenan-induced hypersensitivity was improved by the inhibition of spinal PAR2 using an i.t. treatment with PAR2 antagonist FSLLRY-NH2. In vitro electrophysiological recordings from superficial dorsal horn neurons in acute spinal cord slices prepared 24 h after the induction of peripheral inflammation revealed an increase of the mEPSC and sEPSC frequencies and the amplitude of dorsal root stimulation-evoked EPSCs after a bath application of the PAR2-activating peptide. This PAR2 activation-induced potentiating effect on the EPSCs was attenuated by TRPV1 and protein kinase inhibitions. On the other hand, inhibition of the spinal PAR2 by the antagonist decreased the frequencies of mEPSC and sEPSC and the amplitude of eEPSC, suggesting the activation of spinal PAR2 due to the peripheral inflammation itself. Our findings clearly demonstrate that spinal PAR2 are already activated during the carrageenan-induced peripheral inflammation and enhance the nociceptive excitatory synaptic transmission in the spinal cord and contribute to thermal hyperalgesia. The mechanisms of spinal PAR2 activation under the inflammatory conditions involve protein kinases and TRPV1 activation.

### 3.1. Mechanisms of Spinal PAR2-Mediated Thermal Hyperalgesia

The importance of spinal PAR2 activation for the development of hypersensitivity was demonstrated using an i.t. treatment with protease (thrombin) and PAR AP, while the PAR2-mediated pronociceptive effect was abolished in PAR2-deficient mice [21,28]. Additionally, the hypersensitivity elicited by the intraplantar injection of PAR2 AP was attenuated in TRPV1-deficiente mice [15,16]. Our previous work suggested that spinal PAR2 activation-induced thermal hyperalgesia in naïve rats was mediated by protein kinases and TRPV1 receptors on the central endings of the DRG neurons [34]. Here, we showed that the activation of spinal PAR2 further enhanced the thermal hyperalgesia induced by carrageenan injection in the paw. Additionally, the mechanisms under the inflammatory conditions were similar to the naïve conditions, both involving protein kinases and TRPV1 activation. However, the inhibition of protein kinases was more effective in our current experiments under the inflammatory conditions than in the naive rats [34]. Besides TRPV1, protein kinases in the spinal cord target other key receptors and channels involved in nociception, resulting in hypersensitivity in vivo, especially during neuroinflammation [43]. Thus, the i.t. staurosporine used in our experiments most likely ameliorated the inflammation-induced hyperalgesia also via ways other than the TRPV1 pathway.

Earlier studies indicated that not only peripheral but, also, central TRPV1 activation considerably contributes to the thermal hypersensitivity induced by peripheral inflammation using the CFA-induced chronic model of persistent inflammatory pain [44,45,46,47]. In the carrageenan-induced model of acute inflammatory pain, thermal hyperalgesia did not develop in TRPV1 knockout mice [48,49]. An intrathecal pretreatment with TRPV1 antagonists (AMG 9810) before carrageenan injection significantly attenuated the intraplantar carrageenan-evoked hypersensitivity [50]. In addition, the carrageenan treatment increased the level of TRPV1 in the spinal cord and the capsaicin-evoked release of glutamate in the dorsal horn [51] similar to the CFA model of inflammation [52]. While both the CFA- and carrageenan-induced models lead to the development of thermal hypersensitivity, the time course and mechanisms are not identical [53]. The type of antagonist used and the timing of the treatment may also play important roles in the analgesic effect of the TRPV1 antagonist, whereas the pretreatment [50] could be more effective than the posttreatment. In our experiments, SB 366791 was administered (i.t.) 24 h after the carrageenan injection but 10 min before the PAR2 AP i.t. treatment. It seems plausible that the SB 366791 treatment did not block the already induced changes in the spinal cord dorsal horn triggered by the peripheral nerve activity after the carrageenan injection, whereas the subsequent PAR2 AP treatment-induced hyperalgesia was blocked effectively.

### 3.2. The Effect of Carrageenan on the Basal Spontaneous EPSC Frequency and Synaptic Transmission

In accordance with our earlier findings [25], the basal control frequencies of both the mEPSC and sEPSC recorded in the slices from animals with peripheral inflammation were higher compared with the appropriate controls in our study performed using naive animals [34]. These results indicate that the spontaneous glutamate release, measured by changes in the frequency of both sEPSC and mEPSC (isolated by tetrodotoxin application) in the superficial dorsal horn was increased after the carrageenan-induced inflammation and may lead to the increased activity and excitability of nociceptive dorsal horn neurons.

In agreement with the effect of PAR2 activation in our in vivo experiments, the application of PAR2 AP on the spinal cord slices dissected 24 h after inflammation increased the frequency of the mEPSC and sEPSC and the amplitude of the dorsal root stimulation-evoked EPSC. Activating peptide SLIGKV used in our experiments mimics PAR2 activation by the canonical mechanism involving signaling from the plasma membrane and endosome. A prolonged potentiating effect on the excitatory synaptic transmission could be underlined by the irreversible mechanism of proteolytic PAR2 activation. Cleavage exposes the tethered ligand domain available to interact with the receptor; subsequently, persistent signaling at the plasma membrane and in the endosome occurs [8]. In addition, activated PAR2 undergo desensitization mediated by β-arrestins, presumably through phosphorylation, internalization and signaling to downstream effectors [11,13,14,18,19,54].

Based on the findings indicating the extremely high co-expression of PAR2 and TRPV1 receptors in the DRG neurons, the evidence of their interaction was demonstrated previously not only in the HEK cells but, also, in the DRG neurons [15,16]. Our experiments supported the strong evidence of PKC involvement after PAR2 activation in the spinal endings of nociceptive DRG neurons, leading to the subsequent phosphorylation of TRPV1, which could increase the TRPV1 sensitivity to endogenous agonists and decrease the temperature threshold for the nociceptive response [15,16,25,55,56]. The activation of spinal PAR2 leads also to the increased release of glutamate and neuropeptides in the spinal dorsal horn through the activation of sensitized presynaptic TRPV1 [5,15,34]. This PAR2-induced and TRPV1-mediated mechanism of nociceptive synaptic transmission augmentation in the dorsal horn most likely plays a role also after the intraplantar carrageenan injection. The presynaptic TRPV1-mediated potentiation of glutamatergic input to the lamina I neurons was also recently demonstrated in the CFA-induced inflammatory model [47].

The potentiating effect of PAR2 activation on the sEPSC frequency and eEPSC amplitude was similar in both the naïve [34] and the inflammatory conditions. In contrast to the surprising PAR2 activation-induced TRPV1-mediated inhibitory effect on the mEPSC frequency in the naïve animals [34], after the carrageenan-induced inflammation, the PAR2 activation evoked a robust increase of the mEPSC frequency. While the effect of spinal PAR2 activation on the mEPSC in naïve animals was inhibitory, the resulting behavioral effect was also pronociceptive in naïve conditions. This indicates that the PAR2-mediated increase in the eEPSC and sEPSC better reflects the final effect on the excitatory synaptic transmission and underlies the behavioral effect.

### 3.3. Activation of Spinal PAR2 after Peripheral Inflammation

In this paper, we show for the first time that the intrathecal application of the PAR2 antagonist FSLLRY-NH2 had a substantial antinociceptive effect on carrageenan-induced inflammation and reduced the enhanced activation of dorsal horn neurons demonstrated by recordings of mEPSC, sEPSC and eEPSC. This suggests that peripheral inflammation leads to the endogenous activation of spinal PAR2 receptors that contribute to the spinal mechanisms, leading to increased sensitivity. The involvement of spinal PAR2 activation was demonstrated before in other pathological pain conditions like bone cancer, oxaliplatin-induced neuropathic pain, bladder pain and chronic arthritis [57,58,59,60,61]. We suggest that carrageenan-induced peripheral inflammation triggers an increased activity in primary afferent fibers ending in the dorsal horn and modulates nociceptive signaling among others via the activation of spinal presynaptic PAR2.

## 4. Methods

### 4.1. Statement of Ethical Considerations

All experiments were approved by the Animal Care and Use Committee of the Institute of Physiology CAS, Prague, Czech Republic and were carried out in accordance with the guidelines of the International Association for the Study of Pain and EU Directive 2010/63/EU for animal experiments. All efforts were made to minimize animal suffering, to reduce the number of animals used and to use alternatives to in vivo techniques, if available.

### 4.2. Animal Care and Utilization

Altogether, 72 male Wistar rats (Institute of Physiology, CAS, Prague, Czech Republic) were used in this study. The animals were housed in a temperature-controlled facility at 23 ± 2 °C with free access to food and water and maintained on a 12-h light, 12-h dark cycle and were checked twice a day. All the animals were handled only for a necessary period of time and, throughout the experiment, did not show any signs of stress or illness. Animals were sacrificed at the end of the experiment by deep anesthesia with isoflurane 3% (Forane^®^, Abbott, Barcelona, Spain) or ketamine (150 mg/kg) and xylazine (20 mg/kg), with subsequent medulla interruption and exsanguination. No animal was excluded from the study or sacrificed for disease.

### 4.3. Spinal Cord Slice Preparation

Acute spinal cord slices were prepared from male Wistar rats on postnatal days P21–P23, similar to previously published data [25,62]. After deep anesthesia with 3% isoflurane (Forane^®^, Abbott, Barcelona, Spain), the lumbar spinal cord was removed and immersed in oxygenated ice-cold dissection solution containing (in mM): 95 NaCl, 1.8 KCl, 7 MgSO_4_, 1.2 KH_2_PO_4_, 26 NaHCO_3_, 0.5 CaCl_2_, 25 D-glucose and 50 sucrose. The spinal cord was then fixed to vibratome stage (Leica VT1200S, Wetzlar, Germany) using cyanoacrylate glue in a groove between two agar blocks. Transverse slices 300-μm-thick were cut from the lumbar segment L3–L5, incubated in the dissection solution for 30 min at 33 °C and then stored in a recording solution at room temperature until used for the electrophysiological experiments. The recording solution contained (in mM): 127 NaCl, 1.8 KCl, 1.3 MgSO_4_, 1.2 KH_2_PO_4_, 26 NaHCO_3_, 2.4 CaCl_2_ and 25 D-glucose. For the actual measurement, slices were transferred into a recording chamber continuously perfused with the recording solution at a rate ~2 mL/min. All extracellular solutions were saturated with carbogen (95% O_2_, 5% CO_2_) during the whole process.

### 4.4. Patch-Clamp Recordings

Patch-clamp recordings were made from superficial dorsal horn neurons in laminae I and II (outer) in acute spinal cord slices. Individual neurons were visualized using a differential interference contrast microscope (Leica, DM LFSA, Wetzlar, Germany) equipped with a near infrared-sensitive camera (Hitachi KP-200P, Tokyo, Japan) with a standard TV/video monitor. Patch pipettes were pulled from borosilicate glass tubing with resistances of 3.5–6.0 MΩ when filled with intracellular solution. The intracellular pipette solution contained (in mM): 125 gluconic acid lactone, 15 CsCl, 10 EGTA, 10 HEPES, 1 CaCl_2_, 2 MgATP and 0.5 NaGTP and was adjusted to pH 7.2 with CsOH. Voltage-clamp recordings in the whole-cell configuration were performed with an Axopatch 200B amplifier and Digidata 1440A digitizer (Molecular Devices, Sunnyvale, CA, USA) at room temperature (~23 °C). Recordings were low-pass-filtered at 2 kHz and digitally sampled at 10 kHz. The series resistance of the neurons was routinely compensated by 80% and was monitored during the whole experiment. AMPA receptor-mediated spontaneous, miniature and evoked EPSCs were recorded from neurons clamped at -70 mV in the presence of 10-μM bicuculline and 5-μM strychnine. Miniature EPSCs were distinguished by the addition of 0.5-μM tetrodotoxin to the bath solution. In order to record evoked EPSCs, a dorsal root was stimulated using a suction electrode with glass pipette filled with an extracellular solution using a constant current isolated stimulator (Digitimer DS3, Hertfordshire, England). The intensity of the stimulation was adjusted to evoke a stable EPSC with a 0.5-ms stimulus duration and at least 3x the minimal stimulus current at a frequency of 0.033 Hz.

The experiments started with control recordings (4 min), followed by PAR2 AP (SLIGKV-NH_2_, 100 μM, 4 min) or PAR2 antagonist (FSLLRY-NH_2_, 100 μM, 4 min) application. In the groups where the TRPV1 or PK antagonist was used (SB 366791, 10 μM and staurosporine, 250 nM), it was applied for 4 min after the control recording as a pretreatment and then with PAR2 AP (100 μM) as co-application. The concentration of PAR2 AP was based on the EC_50_ and previously used effective concentration [12,34]. The concentration of SB 366791 was determined from our earlier studies [25,27]. The mEPSC and sEPSC signals were always evaluated during the last two minutes of the specific application. Amplitudes of the evoked EPSCs were recorded every 30 s; the average amplitude of the 4 last evoked currents during the particular application was always used for evaluation of the specific condition. Neurons with capsaicin-sensitive afferent inputs were identified by an increase of EPSC frequency (> 20%), measured after the capsaicin (200 nM) application at the end of each recording protocol.

The software package pCLAMP 10 (Molecular devices, Sunnyvale, CA, USA) was used for data acquisition and subsequent off-line analysis. Data segments of 2-min durations were analyzed for each experimental condition. Only EPSCs with an amplitude of 5 pA or greater (which corresponded to at least twice the recording noise level) were included in the frequency analysis. The same events and data segments were used for the amplitude analysis. Data were normalized as a percentage of the control values (100%) and expressed as means ± standard errors of the mean (SEM). For statistical analyses of significant differences, the Wilcoxon signed rank test, one-way analysis of variance (ANOVA) with the Kruskal-Wallis test or one-way ANOVA followed by the Student-Newman-Keuls (SNK) post-hoc test were used. Detailed information is given in the figure legends. The basal frequencies of mEPSC and sEPSC were compared using a *t*-test. The criterion for statistical significance was *p* < 0.05.

### 4.5. Drug Treatment

All basic chemicals, used for the preparation of dissection, recordings and intracellular solutions, were of analytical grade and purchased from Sigma-Aldrich (Prague, Czech Republic) and Tocris Bioscience (Bristol, UK). Capsaicin, PAR2 AP (SLIGKV-NH_2_), PAR2 NP (VKGILS-NH_2_), PAR2 antagonist FSLLRY-NH2, SB 366791 and staurosporine were dissolved in DMSO, which had a concentration of < 0.1% in the final solution.

### 4.6. Intrathecal Catheter Implantation

The experiments were conducted using adult male Wistar rats (250–300 g). Lumbosacral catheters were implanted between the L4 and L5 vertebrae one week before the experiment. Catheter implantations were performed under brief isoflurane (3%, Forane^®^, Abbott, Barcelona, Spain), followed by ketamine (100 mg/kg) and xylazine (16 mg/kg) anesthesia. The catheters were constructed from polyethylene tubing (PE5) and were fixed with dental cement (Duracryl) to the vertebral bones. The other end of each catheter was fixed to PE10 tubing and externalized on the back of the animal. The positions of the catheters were verified by a dye injection at the end of each experiment. Drugs were applied and flushed (50 or 45 μL) from the catheter by a physiological solution: PAR2 AP and PAR2 NP (8 μg in 10 μL), SB 366791 (0.43 μg in 15 μL), staurosporine (0.014 μg in 15 μL) and PAR2 antagonist FSLLRY-NH_2_ (10 μg in 10 μL). The same vehicle consisting of the physiological solution and < 0.1% DMSO was used for all the i.t. applications. The i.t. treatment with the inactive PAR2 NP was used as a control for the i.t. treatments with active compounds (PAR2 AP, SB 366791 + PAR2 AP, staurosporine + PAR2 AP and PAR2 antagonist) during the behavioral tests. We showed previously that an i.t. application of this vehicle has no effect on the behavioral changes [27,63].

### 4.7. Carrageenan Model of Peripheral Inflammation

Peripheral inflammation of the hind paw was induced 24 h before the i.t. treatment or spinal cord slice preparation. Under brief isoflurane (3%, Forane^®^, Abbott, Barcelona, Spain), an anesthesia intraplantar injection of 20 μL of carrageenan (3%, Sigma-Aldrich, St. Louis, MO, USA) dissolved in a physiological solution was made in both hind paws of 20–22-day-old (40–50 g) rats before spinal cord slices preparation for electrophysiological experiments and 150 μL of carrageenan (3%) into the hind paw of 8–10-week-old (250–350 g) rats used for behavioral testing. The animals were left to recover in their home cages. The inflammation with associated thermal hyperalgesia was fully developed 24 h after carrageenan injection.

### 4.8. Behavioral Tests

The paw withdrawal latency (PWL) to a heat stimulus was tested using a plantar test apparatus (Ugo Basile, Gemonio VA, Italy) with radiant heat applied to the plantar surface of each hind paw. Rats were placed in nonbinding, clear plastic cages on a clear glass plate, elevated to allow the application of a controlled heat source underneath. Each rat was left to adapt to the testing environment for at least 15 min prior to any stimulation. The PWL was measured automatically with the apparatus. Each hind paw was tested 4 times with at least 5 min between the trials, and the mean was calculated. The averaged values from the left and right hind paws in individual animals were then averaged in the experimental groups. Baseline withdrawal latencies were determined in all animals before any experimental procedure.

Data were normalized as a percentage of the control values (100%) and expressed as the means ± SEM. Wilcoxon signed rank test was performed in analyzing the carrageenan effect. Kruskal-Wallis one-way ANOVA was used to compare the differences in each experimental group versus the normalized control before i.t. treatment. One-way ANOVA followed by a Student-Newman-Keuls post-hoc test with multiple comparisons was used to identify the differences between various treatments. The criterion for statistical significance was *p* < 0.05.

## 5. Conclusions

Our study demonstrated that carrageenan-induced paw inflammation elicited a modulation of the nociceptive signaling in the spinal cord dorsal horn, including constitutive PAR2 activation. The contribution of the PAR2 activity at the presynaptic endings of nociceptive DRG neurons in the spinal cord to the hypersensitivity elicited by peripheral inflammation is facilitated by the enhancement of spontaneous and dorsal root stimulation-evoked excitatory synaptic transmissions. The mechanism of the pronociceptive effect induced by PAR2 stimulation involves the activation of spinal protein kinases and TRPV1 receptors. A better understanding of the alterations in nociceptive synaptic transmissions in the spinal cord that occur as a result of peripheral inflammation and underlie the associated hypersensitivity is needed for the development of more efficient pain therapy.

## Data Availability

All relevant data are within the paper.
